# Estrogen Receptor–Phytoestrogen Interactions in Health and Aging: A Review on Estrogen Receptor Vascular Actions with Proof-of-Concept Data

**DOI:** 10.3390/nu18050741

**Published:** 2026-02-26

**Authors:** Bailey Smith, Kailey Myers, Katelyn Nigro, Sujin Bao, Xuan Yu, Guichun Han

**Affiliations:** 1 Department of Biomedical Sciences, Kentucky College of Osteopathic Medicine, University of Pikeville, Pikeville, KY 41501, USA; baileysmith@upike.edu (B.S.); kaileymyers@upike.edu (K.M.); katelynnigro@upike.edu (K.N.); sujinbao@upike.edu (S.B.); 2 Anesthesia-Department Research, Stanford University, Stanford, CA 94305, USA; xyu2018@stanford.edu

**Keywords:** postmenopausal health, estrogen receptors, nutrition, cardiovascular aging, phytoestrogens, GPER, cardiovascular health, sarcopenia, osteoporosis, metabolic dysfunction, endothelial dysfunction

## Abstract

**Background/Objectives**: The menopausal decline in estrogen levels accelerates age-related changes, including visceral adiposity, insulin resistance, sarcopenia, osteoporosis, and endothelial dysfunction. While nutrition independently influences these outcomes, the interactive role of estrogen signaling and nutrient metabolism in healthy aging remains underexplored. This article evaluates these interactions. **Methods**: We conducted a narrative synthesis of studies examining estrogen’s effects on energy balance, adipose regulation, muscle, bone, and cardiovascular health, with an emphasis on estrogen-like nutritional modulators and phytoestrogens. In addition, we present original experimental data from our laboratory investigating sex-specific vascular responses to G protein-coupled estrogen receptor (GPER) activation using functional myography in isolated rat aortic rings from young adult and middle-aged rats (n = 6–8 per group) to assess responses to the GPER agonist G-1 (1.0 μM). **Results**: Literature evidence demonstrates that estrogen supports macronutrient utilization, suppresses adipose inflammation, preserves bone density, and promotes endothelial function. Phytoestrogens may engage estrogen-responsive pathways to mitigate age-related physiological decline. Our original findings show that GPER agonism enhances both contractile and vasodilatory responses in female (*p* < 0.05) but not male rat aortas, providing mechanistic evidence of sex-specific vascular estrogen signaling. These results suggest that dietary phytoestrogens and nutrient-rich dietary patterns may, in part, activate GPER-dependent pathways to support cardiovascular resilience in aging women. **Conclusions**: Estrogen–nutrition interactions are central to metabolic adaptation and healthy aging. Our findings highlight GPER as a functionally resilient pathway in aging vasculature, offering a putative mechanistic link for nutritional modulation. However, direct translation of these findings to human clinical outcomes remains to be established. Precision nutrition strategies targeting GPER represent a promising framework for healthy aging, though large-scale human trials are necessary to confirm these receptor-specific effects.

## 1. Introduction

Aging is associated with a rising prevalence of metabolic, musculoskeletal, and cardiovascular diseases driven by cumulative endocrine, inflammatory, and metabolic alterations [1]. Among endocrine regulators, estrogen plays a central role in maintaining physiological homeostasis across energy metabolism, adipose tissue distribution, skeletal integrity, muscle mass, and vascular function [2,3]. Loss of estrogen during the menopausal transition accelerates visceral fat accumulation, insulin resistance, sarcopenia, bone loss, and endothelial dysfunction beyond changes attributable to chronological aging alone [1,2,4,5,6].

Nutrition is increasingly recognized as one of the major determinants of aging outcomes and chronic disease risk through both direct metabolic actions and endocrine interactions [1,2,7,8]. Dietary patterns, phytoestrogen intake, and gut microbiome composition influence estrogen bioavailability and downstream signaling, particularly during the midlife hormonal transition [8,9,10,11]. Growing evidence suggests that dietary factors interact with estrogen-dependent pathways to support metabolic resilience, cardiovascular health, and longevity in aging women [2,5,7,12].

Several reviews comprehensively examined how estrogen receptor subtypes, ERα, ERβ, and GPER, may differentially respond to nutritional signals and thereby regulate metabolic, musculoskeletal, and vascular aging [8,13,14]. However, much of the existing literature examined estrogen signaling and nutrition independently, but an integrated understanding of the determinants of aging outcomes is missing [8]. This gap has limited the development of precision nutrition strategies aligned with estrogen-dependent signaling across organ systems [11,13].

In this review, we propose that estrogen receptor signaling functions as a central integrator of metabolic, musculoskeletal, and vascular regulation, and that nutritional exposures may selectively modulate these receptor-specific pathways. We hypothesize that non-classical estrogen signaling—particularly through G protein-coupled estrogen receptor (GPER)—represents a mechanistically resilient pathway that may be leveraged for targeted nutritional intervention during female aging. While epidemiological and observational studies suggest associations between dietary patterns and cardiometabolic health in women, clinical trials evaluating phytoestrogens and receptor-specific outcomes have produced inconsistent results. Furthermore, mechanistic studies examining estrogen receptor–nutrient interactions are often conducted in isolation, without integration across organ systems. This gap limits the development of precision nutrition strategies grounded in receptor biology. To address this, we synthesize mechanistic, observational, and interventional evidence while incorporating original experimental data to examine sex-specific vascular responses to GPER activation, thereby advancing a receptor-focused framework for nutrition-informed aging strategies.

## 2. Phytoestrogens ‘Interaction with Estrogen and Healthy Aging: A Background of Phytoestrogens’ Mechanism

As endogenous estrogen levels decline with age, diet-derived estrogenic compounds emerge as a biologically plausible mechanism through which nutritional exposures may modulate estrogen receptor signaling and influence aging trajectories [1,2,8]. Phytoestrogens are a diverse group of plant-derived non-steroidal polyphenolic compounds that exhibit structural and functional similarity to 17β-estradiol, the principal biologically active form of endogenous estrogen, enabling interaction with estrogen receptors [15]. This receptor interaction is mediated by phenolic hydroxyl groups that are spatially arranged to permit binding analogous to 17β-estradiol, although with substantially lower affinity [8]. Based on shared structural features and biological activity, phytoestrogens are broadly classified into isoflavones, lignans, coumestans, and stilbenes, which are widely distributed across plant-based foods including legumes, seeds, whole grains, fruits, vegetables, teas, and cocoa [10,15].

Dietary phytoestrogens are predominantly consumed as glycosylated precursors that require enzymatic deglycosylation prior to intestinal absorption [8,10]. Following ingestion, these compounds undergo extensive biotransformation by intestinal epithelial enzymes and the gut microbiota, generating metabolites with enhanced biological activity and tissue specificity [10,11,16,17,18,19]. Only a small fraction of ingested phytoestrogens enters systemic circulation in unmetabolized form, underscoring the importance of host metabolism and microbiome composition in determining bioavailability and physiological effects [11,16]. This microbial dependency creates significant inter-individual variability, such as the equol-producer phenotype, which may explain the inconsistent clinical outcomes often observed in human studies [10,19].

Phytoestrogens exert their biological actions through interactions with multiple estrogen receptor subtypes, including estrogen receptor α (ERα), estrogen receptor β (ERβ), and the G protein-coupled estrogen receptor (GPER), each of which exhibits distinct cellular localization, signaling dynamics, and tissue distribution [8,16]. While classical nuclear receptors primarily mediate transcriptional responses, GPER initiates rapid non-genomic signaling pathways that influence vascular tone, metabolic regulation, and inflammatory responses [13,14,20]. Consequently, phytoestrogen effects are highly context-dependent, varying according to dose, metabolic conversion, receptor expression patterns, and target tissue [8,11]. However, a notable limitation of the current evidence base is the reliance on experimental models using high-affinity agonists or concentrated doses that may exceed realistic dietary levels, where phytoestrogens likely function as weak modulators [8,11,13,16,17,18,19,20]. The receptor-specific complexity provides a mechanistic foundation for understanding how dietary phytoestrogens may selectively modulate estrogen-dependent pathways during aging [13]. 

Collectively, these receptor-specific signaling pathways illustrate how phytoestrogens engage in both genomic and non-genomic estrogen mechanisms to influence vascular, metabolic, and inflammatory processes. Because phytoestrogen activity depends on dose, metabolic conversion, receptor distribution, and tissue context, physiological responses are inherently variable across individuals and life stages. This mechanistic complexity highlights the need to evaluate human outcome data to determine whether and under what conditions dietary phytoestrogens confer measurable health benefits during the postmenopausal period, particularly considering the translational gap between isolated compounds and whole-food matrices.

## 3. Estrogen and Energy Metabolism

### 3.1. Hormonal Decline and Metabolic Consequences

Estrogen receptor signaling functions as a central regulator of systemic energy homeostasis through coordinated effects on adipose tissue biology, skeletal muscle metabolism, and hepatic substrate handling [2,3]. Across metabolic tissues, estrogen modulates mitochondrial function, substrate partitioning, and inflammatory tone, thereby influencing whole-body metabolic efficiency.

Within adipose tissue, estrogen receptor activation restrains visceral fat expansion by limiting excessive lipolysis and regulating adipocyte differentiation [2,3,20]. Diminished signaling shifts lipid storage toward visceral depots, where elevated free fatty acid flux promotes insulin resistance and ectopic lipid accumulation [20]. Experimental ovariectomy models demonstrate that these alterations occur independent of chronological aging, supporting a direct regulatory role of estrogen in maintaining adipose distribution and metabolic stability [12,17,21]. At the transcriptional level, reduced estrogen receptor activity downregulates genes involved in mitochondrial β-oxidation and biogenesis, impairing fatty acid utilization and reducing oxidative capacity [12,13,14]. The resulting mismatch between lipid availability and mitochondrial function contributes to metabolic inflexibility and adipocyte hypertrophy.

Estrogen also coordinates carbohydrate metabolism by enhancing insulin-mediated glucose uptake and glycogen storage in skeletal muscle and liver [20]. Through receptor-mediated signaling pathways, estrogen improves insulin sensitivity and promotes efficient substrate switching between lipid and glucose oxidation. Attenuation of this signaling disrupts glycemic control and reduces metabolic adaptability, contributing to impaired glucose handling even in the absence of substantial weight gain [20].

In skeletal muscle, estrogen receptor activation supports lean mass maintenance by suppressing protein catabolism and promoting mitochondrial efficiency [4,22]. Reduced signaling shifts muscle protein balance toward net breakdown and diminishes oxidative capacity, changes that compromise metabolic resilience and exacerbate systemic insulin resistance. These coordinated alterations across adipose, hepatic, and skeletal muscle tissues underscore the integrative role of estrogen receptors in maintaining metabolic flexibility.

Collectively, estrogen signaling acts as a metabolic integrator that aligns substrate utilization, mitochondrial function, and inflammatory regulation across tissues. Disruption of this receptor-mediated network alters lipid partitioning, glucose metabolism, and muscle energetics, establishing a physiologic environment characterized by reduced oxidative efficiency and increased metabolic vulnerability.

### 3.2. Estrogen–Macronutrient Crosstalk and Nutritional Modulation of Metabolic Health 

Estrogen coordinates macronutrient metabolism by regulating carbohydrate, lipid, and protein utilization across metabolic tissues, including skeletal muscle, liver, and adipose tissue [2,3,20]. In carbohydrate metabolism, estrogen enhances insulin-mediated glucose uptake and promotes glycogen storage in skeletal muscle and liver, whereas estrogen deficiency contributes to glucose intolerance and reduced metabolic efficiency [20,21]. Progesterone can antagonize these effects by suppressing glucose uptake and altering gluconeogenic output, underscoring the importance of hormonal balance in maintaining glycemic control [20]. 

Lipid metabolism is particularly sensitive to estrogen signaling [2,3]. Estrogen enhances mitochondrial fatty acid oxidation and restrains excessive lipolysis, thereby limiting elevations in circulating free fatty acids [12,20,21]. Reduced estrogen availability disrupts these controls, promoting adipocyte hypertrophy, lipid spillover, and chronic low-grade inflammation [2,3]. Alterations in ERα and ERβ signaling further influence adipogenic and lipolytic pathways through modulation of PPARγ activity, lipid uptake, and mitochondrial function [8,13,14]. 

Protein metabolism is also hormonally regulated [4,20]. Estrogen suppresses skeletal muscle protein catabolism and supports lean mass maintenance, while progesterone promotes protein breakdown, particularly during periods of increased energy demand [1,20,21]. Following menopause, reduced estrogen signaling shifts protein balance toward net catabolism, contributing to sarcopenic trajectories that impair whole-body metabolic health [4,21,22]. 

Within this hormonal context, targeted nutritional strategies provide non-pharmacologic approaches to mitigating estrogen deficiency-related metabolic dysregulation [2,3]. Optimizing caloric distribution with emphasis on plant-derived unsaturated fats, fiber-rich carbohydrates, and adequate protein intake supports lipid oxidation, preserves lean mass, and limits visceral adiposity [7]. Diets enriched in whole foods and unsaturated fats further improve mitochondrial efficiency and attenuate inflammatory signaling within adipose tissue [2,3,12]. 

Dietary phytoestrogens, including soy isoflavones and flaxseed lignans, engage estrogen-sensitive pathways and partially mimic endogenous estrogen effects on metabolism and inflammation [9]. Evidence for metabolic benefits of phytoestrogen intake in postmenopausal women is mixed and context-dependent [8,23]. Randomized controlled trials suggest that soy isoflavone supplementation may be associated with modest but statistically significant improvements in glycemic regulation, including reductions in fasting glucose and insulin levels [21,24,25]. Together, these findings indicate that the metabolic effects of phytoestrogens are generally modest and influenced by dose, baseline metabolic status, and study design [8,23]. When combined with regular physical activity, nutritional strategies that align with estrogen-responsive metabolic pathways may enhance metabolic resilience and reduce cardiometabolic risk across the menopausal transition [12,21,25,26]. 

## 4. Estrogen and Musculoskeletal Aging

### 4.1. Estrogen Decline and Musculoskeletal Aging: Integrated Muscle–Bone Deterioration 

Age-related loss of musculoskeletal mass and function is a major contributor to frailty, disability, and reduced quality of life, with women experiencing a disproportionately accelerated decline following menopause due to reduced estrogen signaling [1,4,26]. Estrogen plays a central role in maintaining skeletal muscle integrity by suppressing apoptotic pathways, supporting anabolic signaling, and preserving neuromuscular function [4,22,26]. Its withdrawal during the menopausal transition accelerates sarcopenia, reductions in muscle mass, and dynapenia, reductions in muscle quality and functional performance, beyond changes attributable to chronological aging alone [1,4,22]. Menopause is associated with progressive loss of lean mass accompanied by increased intramuscular fat infiltration, impairing force generation and metabolic efficiency [4,22,26]. These structural changes manifest clinically as reduced strength, slower gait speed, increased fatigue, and elevated fall risk [1,4]. 

Estrogen is also a critical regulator of bone remodeling, maintaining skeletal integrity through coordinated effects on osteoclast and osteoblast activity [6,26]. Under physiological conditions, estrogen suppresses osteoclastogenesis while promoting osteoblast survival, in part through inhibition of receptor activator of nuclear factor κB ligand (RANKL) signaling and upregulation of osteoprotegerin, thereby limiting bone resorption [6]. Following menopause, reduced estrogen signaling shifts bone remodeling toward a high-turnover, catabolic state in which resorption outpaces formation, resulting in rapid trabecular bone loss and progressive cortical thinning—particularly in the spine and hip [25,26]. Women lose an estimated 20% of bone mineral density within the first 5–10 years after menopause, underscoring the magnitude of estrogen-dependent skeletal vulnerability [6,26]. 

The simultaneous deterioration of skeletal muscle and bone gives rise to osteosarcopenia, a condition characterized by synergistic loss of musculoskeletal integrity [6,26]. Reduced estrogen signaling concurrently accelerates muscle atrophy and bone resorption, producing a compounded risk profile that exceeds the impact of either condition alone [6,26]. Declines in muscle strength impair postural stability and increase fall risk, while diminished bone density heightens susceptibility to fragility fractures [1,26]. This integrated decline explains why postmenopausal women reach functional disability thresholds earlier than men and experience disproportionately higher rates of falls, fractures, and loss of independence [1,6].

Together, these findings highlight estrogen deficiency as a unifying driver of musculoskeletal aging and emphasize the need for coordinated preventive and therapeutic strategies that target both muscle and bone health rather than isolated interventions [1,6,26]. 

### 4.2. Musculoskeletal Nutritional Considerations

Estrogen deficiency induces anabolic resistance within skeletal muscle by reducing sensitivity to dietary amino acids and attenuating mammalian target of rapamycin (mTOR) signaling, a central regulator of muscle protein synthesis [4,27]. As a result, postmenopausal women may require higher protein intakes—approaching ~1.6 g/kg/day—to preserve muscle mass and functional capacity, exceeding standard adult recommendations [26,27]. Protein intake alone, however, is often insufficient to reverse sarcopenic trends in postmenopausal women and should be paired with mechanical loading [4,25,28]. Resistance exercise acts synergistically with dietary amino acids to sensitize the mTOR pathway, which is otherwise blunted by low estrogen [4,27]. This dual intervention promotes myofibrillar protein synthesis more effectively than one strategy in isolation, helping to maintain the muscle-to-fat ratio necessary for metabolic health [1,24,25,26,27,28,29,30].

Adequate leucine intake is particularly important, as leucine directly stimulates mTOR signaling and enhances protein synthetic responses in estrogen-deficient muscle [4,29]. When combined with resistance exercise, targeted nutritional strategies help restore anabolic responsiveness and mitigate musculoskeletal decline [25,29]. These hormone-informed interventions reinforce the importance of integrating nutrition and physical activity to preserve musculoskeletal resilience across aging [1,26].

Human intervention studies provide the most consistent evidence for phytoestrogen benefits in skeletal health among postmenopausal women [30,31]. Meta-analyses of randomized controlled trials show that soy isoflavone supplementation modestly attenuates postmenopausal bone loss and produces small but significant increases in bone mineral density (BMD), particularly at the lumbar spine and femoral neck, over intervention periods of 6–24 months [30]. Earlier pooled analyses similarly reported increases in BMD and reductions in biochemical markers of bone resorption, with effects most pronounced at higher doses (≥75 mg/day) and among postmenopausal populations [32]. However, high-dose phytoestrogen supplementation has potential adverse effects such as bloating and nausea, causing debates on the efficacy of long-term, high-dose isoflavones on thyroid function in sensitive individuals [30,31,32]. Complementing these findings, narrative reviews indicate that isoflavone intake may favorably modulate bone remodeling by suppressing osteoclastic bone resorption while modestly promoting osteoblastic activity and bone formation [31]. Collectively, these findings support a potential role for phytoestrogens in preserving skeletal health after menopause, while highlighting heterogeneity related to dose, formulation, duration, and individual responsiveness [30,31]. 

## 5. Estrogen and Cardiovascular Health

### 5.1. Estrogen Receptor-Mediated Vascular Regulation

Estrogen receptor signaling plays a critical role in maintaining vascular homeostasis through coordinated genomic and non-genomic mechanisms that regulate endothelial function, oxidative balance, and vascular smooth muscle activity [5,20,26]. Across the arterial wall, estrogen receptors modulate nitric oxide bioavailability, inflammatory signaling, and structural remodeling, collectively preserving vascular tone and compliance.

Activation of ERα and ERβ within endothelial cells suppresses adhesion molecule expression, limits inflammatory cell recruitment, and reduces reactive oxygen species production through inhibition of enzymes such as NADPH oxidase [5,20]. These genomic effects are complemented by rapid, non-genomic signaling pathways that stimulate endothelial nitric oxide synthase (eNOS), increasing nitric oxide (NO) production and promoting vasodilation [13,33]. Through these combined pathways, estrogen receptor activity sustains an anti-inflammatory, antioxidant, and vasodilatory vascular phenotype.

Estrogen also regulates the endothelin-1 (ET-1) system, a key determinant of vascular tone [34]. Under physiological conditions, estradiol suppresses ET-1 transcription and attenuates ET-1-mediated vasoconstrictive signaling. Reduced receptor activity shifts this balance toward enhanced ET-1 expression and heightened vasoconstrictor responsiveness, contributing to impaired endothelial function and increased arterial stiffness [26,33,34]. Beyond endothelial regulation, estrogen influences vascular smooth muscle cell proliferation and extracellular matrix composition. Receptor-mediated signaling preserves collagen–elastin balance within the arterial wall, supporting structural integrity and arterial compliance [20]. When these regulatory mechanisms are attenuated, vascular remodeling accelerates, promoting stiffness, oxidative stress, and diminished vasoreactivity.

Together, these receptor-mediated processes illustrate that estrogen signaling functions as a central regulator of vascular tone, redox equilibrium, and structural resilience. Disruption of this coordinated network alters nitric oxide signaling, endothelin balance, and smooth muscle behavior, creating a vascular environment more susceptible to dysfunction and progressive remodeling.

### 5.2. Cardiovascular Nutritional Considerations

Studies examining the cardiovascular effects of phytoestrogens in postmenopausal women suggest potential but inconsistent cardioprotective benefits [35,36]. Several randomized and observational investigations have reported modest improvements in cardiometabolic risk markers following soy or isoflavone consumption, particularly improvements in insulin resistance and glycemic control among postmenopausal women with existing metabolic risk factors such as type 2 diabetes [24,35]. Meta-analyses have also observed improvements in vascular function-related measures, including arterial stiffness and endothelial responsiveness, outcomes that are mechanistically consistent with estrogen-mediated signaling pathways [37,38]. Prospective cohort data further suggest that habitual dietary phytoestrogen intake may be variably associated with cardiovascular disease risk in women, highlighting differences between population-level associations and interventional outcomes [36]. However, other controlled studies have found no significant effects of phytoestrogen supplementation on lipid profiles, blood pressure, or inflammatory markers, particularly in metabolically healthy postmenopausal women or when isoflavones are administered in isolation rather than as part of whole soy foods [35]. Overall, the evidence suggests that phytoestrogens may confer modest cardiovascular benefits under specific physiological and dietary contexts, while underscoring substantial inter-study heterogeneity and the need for more targeted, mechanism-informed research [35,36,37].

## 6. Defining the GPER Role in Aging Vasculature

Estrogen receptors ERα and ERβ play complementary roles by functioning as the primary mediators for the effects of estrogen across organ systems [39,40]. ERα drives cell growth and division in reproductive tissues like the uterus and breasts while ERβ counteracts ERα to prevent overgrowth, thereby maintaining tissue homeostasis [13,14,15,16,39,40]. The balance of these receptors helps maintain vascular function and tissue elasticity [39,40]. After menopause, the number of ERα and ERβ receptors declines, leading to an attenuated vasodilatory response [13,14,40]. In premenopausal women, estrogen binds ERα on endothelial cell surfaces, triggering eNOS to produce Nitric Oxide for vasodilation [13,20,39]. As ERα, the vessel walls become less receptive, resulting in hypertrophic stiffening [8,14,40]. This is compounded by the loss of ERβ, as this receptor is responsible for smooth muscle relaxation [39,40]. The result for post-menopausal women is hypertension and a 4.3 times higher risk of CVD [39]. The decline in ERα and ERβ receptors also leads to tissue-specific atrophy, affecting circulatory health, which can manifest as temperature dysregulation such as hot flashes [16,17,18,19,20,39,40]. 

To provide a functional context for the mechanisms discussed in preceding sections, we present a focused series of experiments examining GPER’s role in aging vasculature. We sought to determine whether G-protein-coupled estrogen receptor (GPER) signaling represents a female protective mechanism that confers resistance to vascular dysfunction during the premenopausal period by regulating endothelial and smooth muscle control of vascular tone [16,37,38,39,40,41]. Prior genetic and pharmacological evidence demonstrates that loss of GPER abolishes normal sex differences in arterial stiffness and endothelial function and increases susceptibility to endothelin-1 (ET-1)-mediated vasoconstriction in females [13,34]. Sex-dependent regulation of the renal ET-1 system by GPER further supports conserved GPER–ET-1 interactions across vascular beds [13,14]. Building on this evidence, the translational aim of this study was to define how GPER modulates acute vascular tone and whether it shifts the balance between ET-1-mediated constriction and acetylcholine-induced endothelial dilation in a sex-specific manner using a rat aorta model. These experiments were designed to generate foundational mechanistic insights relevant to cardiovascular risk stratification and prevention in postmenopausal women [5,29,34,42]. 

**Figure 1:**
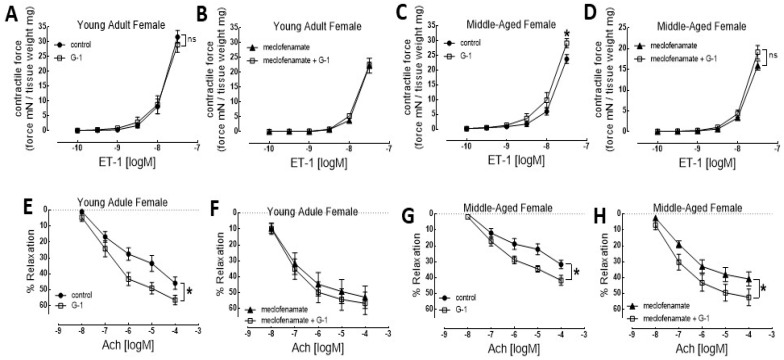
GPER agonist G-1 enhances the ET-1-induced contractile response and ACh-induced endothelial-dependent relaxation of female rat aortic rings. (**A**–**D**) Concentration–response relationship for ET-1-induced aortic contraction: with/without G-1 (1.0 μM) or with meclofenamate (1.0 μM)/and G-1 (1.0 μM); (**A**,**B**) young adult females, (**C**,**D**) middle-aged females. (**E**–**H**) Concentration–response relationship for ACh-induced aortic relaxation: with/without G-1 (1.0 μM) or with meclofenamate (1.0 μM)/and G-1 (1.0 μM); (**E**,**F**) young adult females, (**G**,**H**) middle-aged females. (**A**–**D**) Each point represents the mean developed contractile force ± SEM. Developed tension was the force generated by artery rings normalized to arterial dry weight (mN/mg). (**E**–**H**) Each point represents the mean relaxation response ± SEM. * *p* < 0.05, using a two-way ANOVA.

**Figure 2:**
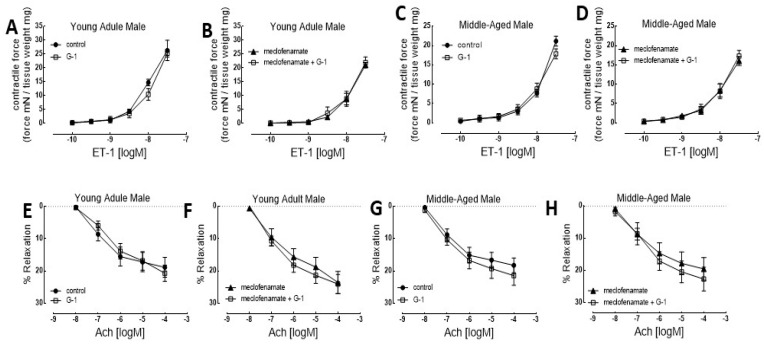
G-1 had no effect on ET-1-induced contractile response and ACh-induced endothelial-dependent relaxation of male rat aortic rings. (**A**–**D**) Concentration–response relationship for ET-1-induced aortic contraction: with/without G-1 (1.0 μM) or with meclofenamate (1.0 μM)/and G-1 (1.0 μM); (**A**,**B**) young adult males, (**C**,**D**) middle-aged males. (**E**–**H**) Concentration–response relationship for ACh-induced aortic relaxation: with/without G-1 (1.0 μM) or with meclofenamate (1.0 μM)/and G-1 (1.0 μM); (**E**,**F**) young adult males, (**G**,**H**) middle-aged males. (**A**–**D**) Each point represents the mean developed contractile force ± SEM. Developed tension was the force generated by artery rings normalized to arterial dry weight (mN/mg). (**E**–**H**) Each point represents the mean relaxation response ± SEM. There are no statistical differences among the paired groups.

In our research, the rat thoracic aorta was selected as a translational model due to its well-characterized endothelial signaling pathways and conservation of estrogen receptor-mediated vascular mechanisms relevant to human physiology. Both young adult and middle-aged male and female rats were studied to assess sex- and age-dependent effects (Figure 1 and Figure 2). Isolated aortic rings were mounted for functional myography to generate concentration–response curves for vasoconstriction using ET-1 and for endothelium-dependent relaxation using acetylcholine (ACh). GPER signaling was selectively activated using the agonist G-1. Meclofenamate was employed to inhibit cyclooxygenase activity, and L-NAME was used to inhibit endothelial nitric oxide synthase (eNOS), allowing interrogation of prostanoid- and nitric oxide-dependent mechanisms. GPER activation produced a sex-exclusive effect on vascular function. Based on our data, we performed a secondary data analysis from a sex-specific perspective and plotted the functional graphs for both female (Figure 1) and male (Figure 2) groups.

The pretreatment of the female young adult aortic rings with G-1 did not change the contractile response to ET-1 (Figure 1A). In another set of experiments, the aortic rings were pre-incubated with meclofenamate (1.0 μM). The addition of G-1 (1.0 μM) in the meclofenamate pretreatment of the aortic rings did not have an effect on ET-1-induced contraction (Figure 1B). In contrast, in experiments with middle-aged female rat aortic rings, G-1 (1.0 μM) pretreatment significantly enhanced ET-1-induced aorta contraction (Figure 1C); pre-incubation of aortic rings with meclofenamate (1.0 μM) attenuated the ET-1-induced contraction response compared to the young adult group, which was blunted by the addition of G-1 (1.0 μM), thus bringing the contractile responses of these two groups closer (Figure 1D). 

As expected, G-1 (1.0 μM) pretreatment enhanced the ACh-induced relaxation response of the aortic rings isolated from young adult and middle-aged female rats (Figure 1E,G). Adding G-1 (1.0 μM) together with meclofenamate (1.0 μM) in the pretreatment of the aortic rings did not have further effects on ACh-induced aorta relaxation in young adult rats but further increased the relaxation effect in middle-aged rats (Figure 1F,H). These data suggest that GPER activation normalizes the compromised contractile response in the aorta isolated from middle-aged female rats and enhances the ACh-induced aorta endothelial-dependent relaxation response.

We found that G-1 pretreatment had no effect on the ET-1-induced concentration response curve (Figure 2A) in the aortic rings of young adult male rats. Addition of G-1 (1.0 μM) to meclofenamate (1.0 μM) in the pretreatment of the aortic rings of young adult male rats did not have additional effects on the ET-1-induced contraction (Figure 2B). Similarly, G-1 pretreatment did not have any significant effect on the concentration response curve of ET-1 in the aortic rings of middle-aged male rats (Figure 2C). Addition of G-1 to the meclofenamate pretreatment did not have any effect (Figure 2D).

In addition, G-1 pretreatment had no effect on ACh-induced relaxation of the aortic rings of both young adult and middle-aged male rats (Figure 2E,G). Likewise, meclofenamate pretreatment did not have any effect on the Ach concentration–response curves either (Figure 2F,H). These data suggest that GPER activation has no remarkable effect on the ET-1-induced contraction or ACh-induced relaxation in young adult and middle-aged male rats.

In summary, in both young adult and middle-aged female rat aortas, G-1 significantly enhanced ET-1-induced contractile responses and augmented ACh-mediated endothelium-dependent relaxation. In contrast, G-1 produced no significant changes in ET-1- or ACh-mediated responses in male rat aortas at either age. Notably, the magnitude of the GPER-mediated response was preserved in middle-aged females, indicating sustained GPER functionality throughout reproductive aging.

This study provides novel mechanistic evidence that GPER is a central mediator of sex-specific vascular regulation. In females, GPER activation presumably enhanced NO-dependent endothelial dilation while modulating ET-1-mediated constriction, indicating coordinated regulation of vasodilatory and vasoconstrictor pathways. The absence of a comparable response in males, probably due to baseline oxidative stress and nitric oxide scavenging, may limit estrogen-dependent signaling in the male vasculature. 

## 7. Summary of Estrogen Receptor Actions in Aging Vasculature

**Figure 3:**
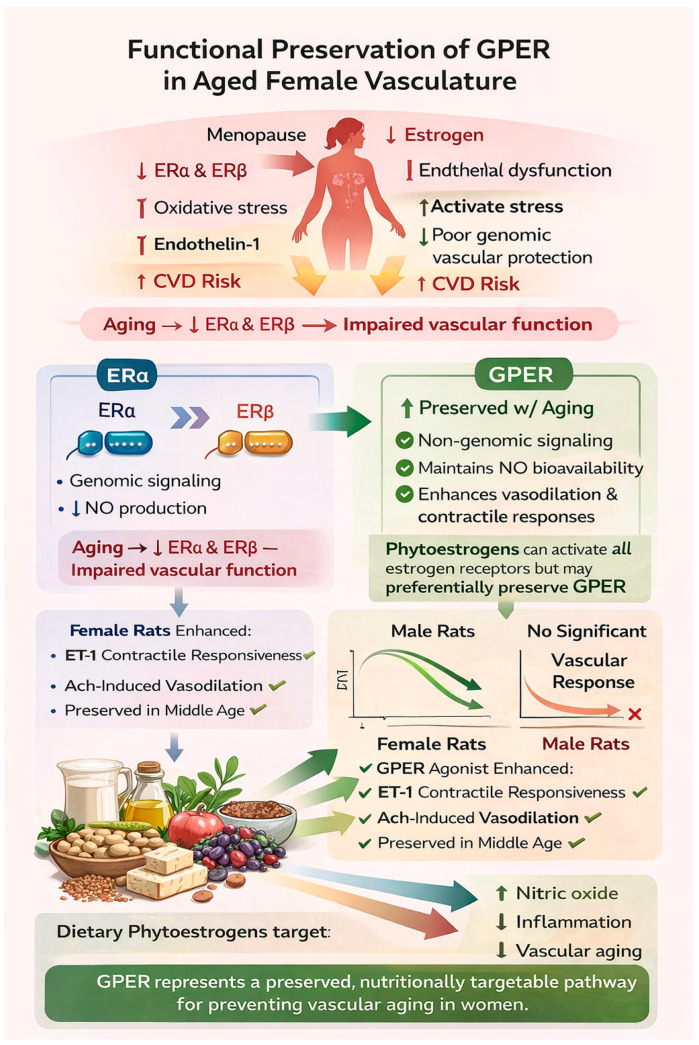
Graphical summary of the estrogen receptor and dietary phytoestrogen interactions in vascular aging. This schematic illustrates how menopause and aging reduce estrogen levels and genomic signaling through ERα and ERβ, contributing to endothelial dysfunction, oxidative stress, and increased cardiovascular disease (CVD) risk. In contrast, G protein–coupled estrogen receptor (GPER) signaling remains relatively preserved with aging and supports non-genomic vascular protection, including maintenance of nitric oxide bioavailability, enhanced vasodilation, and improved contractile responses. Experimental findings demonstrate that GPER-mediated vascular responses are maintained in female but not male rats, particularly in middle age. Dietary phytoestrogens may activate multiple estrogen receptors but appear to preferentially preserve GPER signaling, suggesting a potential therapeutic strategy to reduce vascular inflammation, improve endothelial function, and mitigate vascular aging in women. This figure was generated using ChatGPT (Version 5.2) and modified by the authors.

In summary, based on the available information and our findings, the broader framework of vascular aging and estrogen signaling can be integrated and illustrated as shown in the graph in Figure 3.

With menopause and aging, declining estrogen reduces activation of classical nuclear estrogen receptors (ERα and ERβ), leading to impaired genomic regulation of endothelial function, increased oxidative stress, reduced nitric oxide (NO) bioavailability, and heightened cardiovascular disease risk. In contrast, our data demonstrate that G protein-coupled estrogen receptor (GPER) signaling remains functionally preserved in aged female vasculature. GPER activation promotes rapid, non-genomic signaling through pathways such as PI3K/Akt and eNOS phosphorylation, thereby maintaining NO production and vascular reactivity. Consistent with this mechanism, GPER agonism enhanced acetylcholine-mediated vasodilation and modulated endothelin-1-mediated contractile responses in female but not male vessels. These findings suggest that dietary phytoestrogens may help preserve endothelial function by sustaining GPER-dependent signaling despite estrogen loss, positioning GPER as a nutritionally targetable pathway to mitigate vascular aging and cardiovascular risk in postmenopausal women.

## 8. Discussion

While the transition to menopause is known to accelerate cardiovascular risk through both metabolic and vascular shifts, the specific role of non-classical receptors has been under-explored. Our finding that GPER signaling is functionally preserved in middle-aged female rat aortas (Figure 1) provides a mechanistic bridge suggesting that even as endogenous 17β-estradiol levels decline, the GPER pathway remains viable for intervention. From a translational perspective, preservation of GPER signaling in middle-aged females may buffer against early vascular aging and delay the onset of endothelial dysfunction. Attenuation of GPER activity following menopause may therefore contribute to the rapid rise in cardiovascular disease risk observed in postmenopausal women. These findings position GPER as a promising sex-specific therapeutic target for preventing vascular decline. 

Nutritional modulation represents a complementary translational strategy for supporting GPER-mediated vascular protection. Dietary phytoestrogens may activate GPER-dependent signaling pathways with fewer systemic effects than traditional estrogen therapy. The functional preservation of GPER, as seen in Figure 1, offers a biological rationale for targeted nutritional strategies. Dietary phytoestrogens have structural similarities to 17β-estradiol and can interact with GPER, suggesting they may serve as low-affinity agonists that maintain vascular tone in the absence of high endogenous estrogen. Our data shows that GPER activation enhances NO-dependent dilation in females, supporting the theory that antioxidant-rich patterns like the Mediterranean diet may work synergistically with phytoestrogens to preserve nitric oxide bioavailability.

Clinical translation of nutritional strategies remains complex as seen by conflicting or null results observed in several large-scale human trials [33,35]. These discrepancies may be explained by factors that expand beyond receptor binding. The “Equol Producer” hypothesis suggests that vascular benefits of soy isoflavones are contingent upon the gut microbiome’s ability to metabolize daidzein into equol, a metabolite with higher estrogenic potency; approximately 50–70% of Western populations lack this metabolic capacity, which may decrease the observed efficacy in broad clinical studies [10,17,36]. Additionally, the window of opportunity for nutritional or hormonal intervention is narrow, so interventions initiated after the menopausal transition may find vasculature with extensive remodeling, rendering it less responsive to GPER-mediated vasodilation [5,33]. Finally, the distinction between whole-food matrices and isolated supplements is an important variance. Clinical trials often use purified isoflavones, which lack the synergistic polyphenols and fibers found in a Mediterranean diet that are known to increase nitric oxide bioavailability [35]. Addressing these variables is essential for moving towards a more precise nutritional framework for aging women.

It is important to note that while this study emphasizes the role of GPER, the receptors ERα and ERβ remain vital to vascular homeostasis [20]. ERα typically mediates the long-term genomic effects of estrogen on the vessel wall, such as the regulation of eNOS expression, while ERβ has been implicated in the attenuation of vascular smooth muscle cell proliferation [13,34]. The functional preservation of GPER observed in our rat model may represent a compensatory mechanism that becomes more prominent as the classical ER signaling wanes during early stages of reproductive aging [41]. Therefore, nutritional strategies should use dietary patterns that support the distinct but overlapping pathways of ERα, ERβ, and GPER [43].

Several limitations should be considered when interpreting these findings. First, the experimental data were generated using an isolated rat aorta model, which allows precise mechanistic interrogation of vascular signaling but may not fully capture systemic, tissue-specific, or neurohumoral interactions present in vivo. Second, while the rat model provides valuable translational insight into conserved estrogen receptor pathways, species differences in estrogen metabolism, receptor distribution, and dietary phytoestrogen bioavailability may limit direct extrapolation to human physiology. Specifically, the G-1 concentrations used in our experimental model facilitate the isolation of signaling pathways but may exceed the bioavailability achievable through realistic dietary phytoestrogen intake. Third, the narrative synthesis approach, while comprehensive, lacks formal search strings, strict inclusion/exclusion criteria, and risk-of-bias assessments characteristic of systematic reviews. The inherent subjectivity means that the quality analysis of cited studies was not quantified, potentially influencing the consistency of the evidence presented. Furthermore, the review integrates disparate data types that range from in vitro molecular studies and animal models to human observational cohorts and clinical trials. These levels of evidence can present conflicting results. Although this study demonstrates sex-specific vascular responses to GPER activation, it does not directly establish causal links between dietary phytoestrogens and GPER signaling in humans. While phytoestrogens like genistein are known to bind GPER with high affinity in vitro, clinical studies have yet to isolate GPER-specific effects from those mediated by ERα and ERβ. Finally, the narrative synthesis approach, while comprehensive, is inherently dependent on the scope and quality of available literature.

Future studies incorporating in vivo models, longitudinal human data, and controlled dietary interventions will be necessary to further define the clinical relevance of estrogen–nutrient–GPER interactions in aging populations as a whole, rather than focusing on postmenopausal women. Additional research should prioritize the integration of technologies such as transcriptomics and proteomics to map the GPER–nutrient interactions and clarify how specific dietary ligands trigger non-genomic signaling compared to classical Er pathways in human endothelium. Concurrently, the role of the estrobolome should be investigated to determine how individual variations in microbial composition influence the conversion of dietary precursors into GPER-active metabolites. Finally, there is a need to validate mechanistic findings through human clinical trials that utilize selective, food-derived GPER ligands while accounting for windows of opportunity and diverse ethnic backgrounds. By integrating genetic polymorphisms, lifestyle variables, and habitual dietary patterns, future research will fulfill the ultimate goal of providing a sex-specific approach to promoting vascular resilience and reducing cardiometabolic disease risk.

## 9. Conclusions

Estrogen receptor signaling coordinates metabolic, musculoskeletal, and vascular regulation through interconnected genomic and non-genomic pathways [2,5,20,37]. This review integrates evidence demonstrating that nutritional exposures—including dietary patterns and phytoestrogens—interact with estrogen receptor networks across tissues to influence substrate utilization, skeletal integrity, and vascular tone [8,13,14].

By incorporating original experimental data, we provide mechanistic evidence that G protein-coupled estrogen receptor (GPER) signaling contributes to sex-specific regulation of vascular function and remains functionally responsive during reproductive aging [13,41]. These findings highlight GPER as a potential mechanistic bridge linking estrogen biology and nutritional modulation.

Collectively, this work advances a systems-level framework in which dietary strategies may be aligned with receptor-specific signaling pathways rather than relying solely on systemic hormone replacement approaches [11,43]. While further clinical validation is necessary to define receptor-targeted nutritional effects in humans, integration of estrogen receptor biology with precision nutrition offers a promising direction for supporting vascular resilience and metabolic health across aging.

## Data Availability

No new data were created or analyzed in this study.

## Abbreviations

The following abbreviations are used in this manuscript:

GPER	G-Protein-Coupled Estrogen Receptor
ERα	Estrogen Receptor α
ERβ	Estrogen Receptor β
LDL-C	Low-Density Lipoprotein C
RANKL	Receptor Activator of Nuclear Factor κB Ligand
MTOR	Mammalian Target of Rapamycin
BMD	Bone Mineral Density
CVD	Cardiovascular Disease
NO	Nitric Oxide
ET-1	Endothelin-1
ENOS	Endothelial Nitric Oxide Synthase
ACh	Acetylcholine
μΜ	Micrometer
mN/mg	Millinewton/Milligram
